# Persistent deficits in hippocampal synaptic plasticity accompany losses of hippocampus-dependent memory in a rodent model of psychosis

**DOI:** 10.3389/fnint.2013.00012

**Published:** 2013-03-15

**Authors:** Valentina Wiescholleck, Denise Manahan-Vaughan

**Affiliations:** ^1^Department of Neurophysiology, Medical Faculty, Ruhr University BochumBochum, Germany; ^2^International Graduate School of Neuroscience, Ruhr University BochumBochum, Germany

**Keywords:** dentate gyrus, long-term potentiation, MK801, schizophrenia, *in vivo*

## Abstract

Irreversible *N*-methyl-D-aspartate receptor (NMDAR) antagonism is known to provoke symptoms of psychosis and schizophrenia in healthy humans. NMDAR hypofunction is believed to play a central role in the pathophysiology of both disorders and in an animal model of psychosis, that is based on irreversible antagonism of NMDARs, pronounced deficits in hippocampal synaptic plasticity have been reported shortly after antagonist treatment. Here, we examined the long-term consequences for long-term potentiation (LTP) of a single acute treatment with an irreversible antagonist and investigated whether deficits are associated with memory impairments. The ability to express LTP at the perforant pathway – dentate gyrus synapse, as well as object recognition memory was assessed 1, 2, 3, and 4 weeks after a single treatment of the antagonist, MK801. Here, LTP in freely behaving rats was significantly impaired at all time-points compared to control LTP before treatment. Object recognition memory was also significantly poorer in MK801-treated compared to vehicle-treated animals for several weeks after treatment. Histological analysis revealed no changes in brain tissue. Taken together, these data support that acute treatment with an irreversible NMDAR-antagonist persistently impairs hippocampal functioning on behavioral, as well as synaptic levels. The long-term deficits in synaptic plasticity may underlie the cognitive impairments that are associated with schizophrenia-spectrum disorders.

## INTRODUCTION

The uncompetitive *N*-methyl-D-aspartate (NMDA) receptor antagonist, MK801, is frequently used in neuroscientific research. Its unique kinetics and ability to induce all kinds of schizophrenia symptoms in humans (Krystal et al., 1994; Lahti et al., 2001) has promoted it into one of the drugs of choice in modeling schizophrenia in animals (Rujescu et al., 2006). Acute treatment with MK801 is believed to efficiently mimic a single acute psychosis-like episode in rats that may emulate first-episode psychosis (Kehrer et al., 2007; Wöhrl et al., 2007; Manahan-Vaughan et al., 2008a). Despite its immediate effects, however, it also induces long-lasting dysfunctions, specifically within the cognitive domain. For example, long-lasting impairments in spatial learning in rodents after a single MK801-treatment that persisted for up to months have been reported (Wozniak et al., 1996; Lukoyanov and Paula-Barbosa, 2000; Manahan-Vaughan et al., 2008a,b). Furthermore, it has been shown that a single injection of MK801 to rats results in short-lasting transient behavioral aspects of psychosis-related behavior that may emulate first-episode psychosis (Wöhrl et al., 2007; Manahan-Vaughan et al., 2008a,b).

All of the abovementioned effects are hippocampus-related. Indeed, the hippocampus is one of the brain structures that is most severely affected in schizophrenia pathology. The hippocampus is therefore considered to be a significant element in the development of schizophrenia (Harrison, 2004). NMDA receptor hypofunction is proposed to contribute importantly to the development and progress of schizophrenia (Lindsley et al., 2006). A key question in this possibility is whether the prolonged deficits in hippocampus-dependent memory that are triggered by treatment with an irreversible NMDAR-antagonist, such as MK801, are accompanied by deficits in synaptic plasticity. In the present study, we explored whether the loss of long-term potentiation (LTP) triggered by NMDAR antagonism in this animal model of psychosis persists for long periods, as a clue to the substrate for disturbed cognition as the disease develops. In line with this, we also evaluated cognitive behavior.

Our main goal was to gain insight into the long-term pathophysiology that might develop as a consequence of an acute psychosis-like episode. We found enduring deficits in the ability to express hippocampal LTP 1, 2, 3, and 4 weeks after a single treatment with MK801. Object recognition memory was also impaired for several weeks. Thus, a single treatment with MK801 impairs hippocampal functioning in a long-lasting way on behavioral, as well as synaptic levels, whereby the long-term deficits in synaptic plasticity may underlie the cognitive impairments.

The persistent change in hippocampal functioning after an acute MK801-induced psychosis-like event may be involved into the mechanisms that contribute to the progression of first-episode psychosis into chronic schizophrenia. We hypothesize that the enduring long-term deficit in the ability to express synaptic plasticity and to engage in object recognition memory that occurs in MK801-treated rats may reflect chronic changes in hippocampal function related to psychosis and that the disturbance in synaptic plasticity may contribute to the increased vulnerability of the brain for further schizophreniform episodes.

## MATERIALS AND METHODS

### ETHICAL STANDARDS

The present study was carried out in accordance with the European Communities Council Directive of September 22^nd^, 2010 (2010/63/EU) for care of laboratory animals and after approval of the local ethic committee (senate of Berlin or Bezirksamt Arnsberg). All efforts were made to reduce the number of animals used.

### EXPERIMENTAL ANIMALS

Seven- to eight-week-old male Wistar rats (Charles River, Germany), were housed on a 12-h light/12-h dark cycle (lights on at 07:00 AM) for at least 1 week after their arrival in the animal facility before treatment or surgery.

#### Surgical implantations

Animals were anesthetized (52mg/kg pentobarbital via intraperitoneal injection, i.p.) and underwent chronic implantation of electrodes in the granule cell layer of dentate gyrus (DG) that is part of the hippocampal formation. A monopolar recording electrode (1 mm diameter, 3.1 mm posterior to bregma, 1.9 mm lateral to the midline) and a bipolar stimulating electrode in the perforant pathway (1 mm diameter, 6.9 mm posterior to bregma, 4.1 mm lateral to the midline) were inserted. The animals were allowed between 7 and 10 days to recover from surgery before experiments were conducted.

#### Evoked field potential recordings

Throughout all experiments, the animals could move freely within the recording chamber (40 cm × 40 cm × 40 cm) and had free access to food and water. For acclimatization the animals were transferred to the experiment room 1 day in advance. The implanted electrodes were connected through a head stage by a flexible cable and a swivel connector to the stimulation unit and amplifier. Recordings were stored on a personal computer.

Responses were evoked by stimulating at low frequency (0.025 Hz, 0.2 ms stimulus duration, 10,000 Hz sample rate). For each time-point, five evoked responses were averaged. DG population spike (PS) amplitude, as well as field excitatory postsynaptic potential (fEPSP) slope were monitored: PS amplitude reflects summated action potentials from the somatic layer of granule cells in the DG, whereas alterations of fEPSP indicate dendritic changes. Each experiment started with an input-output (i/o) curve (maximal stimulation 900 μA) to determine the stimulus intensity required to elicit a PS that was of 40% of the maximum obtained in the i/o curve. The i/o-curves between vehicle- and MK801-treated animals did not differ at any time-point after treatment. To ensure stability of recordings and to assess basal synaptic transmission, all animals were tested in a baseline experiment first, where only test-pulse stimulation was applied. LTP was induced by high-frequency stimulation (HFS; 10 bursts of 15 pulses at 200 Hz with 10 s interburst interval) and was recorded 1 week before (pretreatment LTP control) and 1, 2, 3, and 4 weeks after MK801- or vehicle-treatment. PS and fEPSP slope values for pretreatment LTP did not differ significantly in between the two experimental groups. At the beginning of each LTP experiment baseline PS amplitude was obtained by averaging the response to stimulation (five sweeps at 40 s intervals), every 5 min over a period of 60 min. At this point HFS was given, and three additional measurements at 5 min intervals were taken, followed by recordings at 15 min intervals for 24 h.

#### Histology

At the end of the electrophysiological study, brains were removed and histological verification of electrodes and cannula localization was carried out. Brain sections (16 μm) were stained according to the Nissl method using 1% toluidine blue and then examined using a light microscope. Brains, in which an incorrect electrode or cannula localization was found, were discarded from the study.

#### Quantitative analysis of neuron numbers

Male Wistar rats (7–8 weeks old) were treated with either MK801 (mg/kg) or saline. 4 weeks after injection with vehicle (*n* = 5) or MK801 (*n* = 5), the animals underwent cardiac perfusion to fix the brain tissue, as described elsewhere (Naie et al., 2006). Fixed brains were removed immediately after perfusion and sliced coronally. Brain sections (30 μm) were stained according to the Nissl method using 1% toluidine blue. Image acquisition was performed using a light microscope (Zeiss Axioskop, Zeiss, Germany) with a 100 × objective. Plane -5.3, with respect to Bregma (coordinates from Paxinos and Watson, 2007), was considered representative for the three regions of interest (ROIs) – retrosplenial cortex (RSC; layer IV), DG (somatic layer of the upper blade) and entorhinal cortex (EC; layer II) within each hemisphere as indicated in **Figure 5A**. Cell counting of viable neurons within predefined areas of the same size (750 μm × 1000 μm) was carried out using the Image J software by a treatment-blind investigator.

### BEHAVIORAL EXPERIMENTS

#### Open field

The open field test was performed 1 week after MK801 or vehicle treatment in order to assess general locomotor activity of both groups. For this, the animal was placed in an empty gray square polyvinyl chloride arena (80 cm × 80 cm × 80 cm) for 5 min. The light intensity was kept constantly low (5 lux). An automated TSE VideoMot2-Video activity tracking system (TSE Systems, Bad Homburg, Germany) tracked the animal's path. “Total distance traveled” was assessed as an indicator of locomotor activity and “% of time spent in center” was assessed as an indicator for anxiety-related behavior.

#### Object recognition task

The object recognition task (ORT) was performed as described elsewhere (Prickaerts et al., 2002). The apparatus consisted of a gray square polyvinyl chloride arena (80 cm × 80 cm × 80 cm). The test was performed with a constant light intensity of 18 lux. Two different kinds of objects were used: a black polyvinyl rectangle tower (9 cm × 11 cm × 23 cm) and a transparent glass cylinder (10 cm diameter, 30 cm height). The objects could not be displaced by the animals, as they were weighted down (internally) with sand or stones.

Three habituation sessions preceded the test, in which the animals were allowed to explore the empty arena for 5 min each on three preceding days. The testing was carried out directly after the last habituation session. The testing comprised two trials of 3 min duration each. A rat was always placed into arena facing the middle of the front wall. During the first trial (T1) an animal was allowed to explore two identical objects, which were placed symmetrically 20 cm away from the walls. After T1 the rat was put back into its home cage. 4 h later the second trial (T2) took place, in which the rat was placed back into the arena and exposed to a familiar and a novel object. The testing session was videotaped. The times spent exploring each object during T1 and T2 were scored manually. Exploration was defined as directing the nose to the object at a distance of no more than 2 cm and/or touching the object with the nose. Sitting on the object was not considered exploratory behavior. In order to avoid the presence of olfactory cues the objects and the arena were always thoroughly cleaned with 70% ethanol and then washed with water before each trial. Furthermore, as the objects were available in triplicate, neither of the two identical objects from T1 had to be reused in T2. All combinations and locations of objects were used in a balanced manner to reduce potential biases, such as preferences for particular locations or objects. The testing order was determined randomly. Rats that did not explore the objects at all in any trial were removed from analysis.

#### Compounds and drug treatment

The NMDA receptor antagonist [+]-5-methyl-10,11-dihydro-5Hdibenzo-[a,d]-cyclohepten-5,10-imine hydrogen maleate (MK801, Tocris, Germany) was dissolved in 0.9% physiological saline. MK801 (5 mg/kg) or vehicle (10 ml/kg) were injected intraperitoneally (i.p.) 7 days before commencement of first experiments. The concentration of MK801 was chosen in accordance with previous studies (Wöhrl et al., 2007; Manahan-Vaughan et al., 2008a,b), in which the same dose proved to be effective in inducing long-lasting effects. A single high-dose treatment, as opposed to chronic low-dose treatment, was chosen in order to model exclusively the very first acute psychosis-related experience. Directly after injection, acute transient psychosis-like behaviors (locomotion, ataxia, and stereotypy) were scored as described elsewhere (Wöhrl et al., 2007) in order to evaluate the effectiveness of the treatment.

### DATA ANALYSIS

In all electrophysiological experiments, data were expressed as mean % pre-injection values ± standard error of the mean. ANOVA with repeated measures was used to evaluate differences between pretreatment control experiments and experiments after MK801 or vehicle application. Therefore, all values after stimulation (HFS) were compared. In the open field study, one-way ANOVA was used to assess the differences in total distance moved and in percentage of time spent in center between the controls and MK801-treated animals. In the ORT, percentage of total exploration time for each object during T1 and T2 were calculated. *T*-test for independent samples was used in order to analyze time spent exploring the familiar as compared to the new object for every time-point. Differences in total exploration times in T1 and T2 were analyzed via a one-way ANOVA. Statistical analysis was performed using the SPSS software (version 19). Cell counts have been analyzed using a two-way ANOVA (treatment × area). The level of significance was set at *p* < 0.05.

## RESULTS

### BASAL SYNAPTIC TRANSMISSION IS NOT ALTERED 1 WEEK AFTER ACUTE TREATMENT WITH AN IRREVERSIBLE NMDAR ANTAGONIST

**FIGURE 1:**
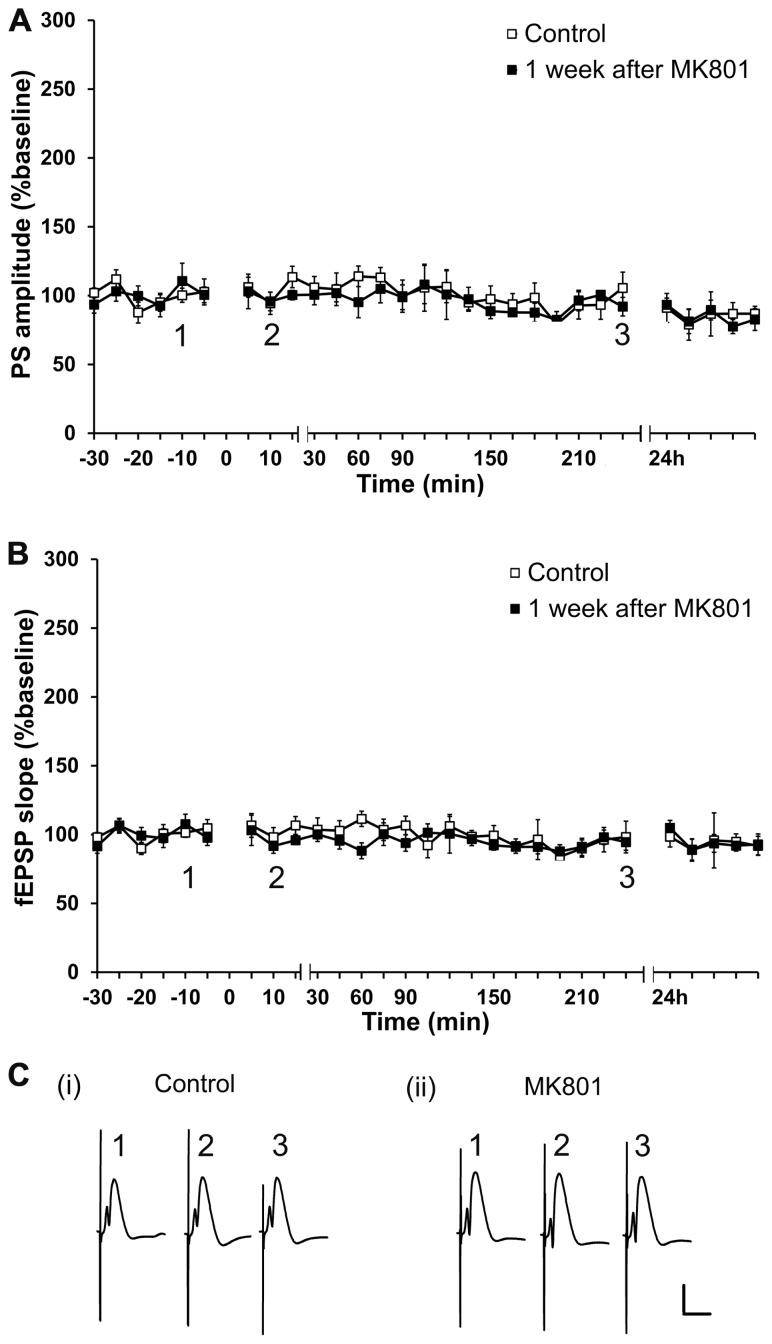
**Acute treatment with an irreversible NMDAR antagonist elicits no lasting effects on basal synaptic transmission.** Basal synaptic transmission is unaffected 1 week after MK801-treatment. No differences in PS amplitude **(A)** or in fEPSP **(B)** occur 1 week after a single MK801-injection (black squares, *n* = 6) as compared to control baseline (white squares, *n* = 6) in response to test-pulse stimulation. Line breaks on the *x*-axis indicate change in time-scale.** (C)** Original analog traces show field potentials evoked from the dentate gyrus during (i) test-pulse stimulation before MK801-treatment and (ii) test-pulse stimulation 1 week after MK801-treatment. Vertical scale-bar corresponds to 5 mV, horizontal scale-bar to 10 ms.

Firstly, possible long-term effects of a single MK801-injection on basal synaptic transmission were assessed. To this end, experiments in which test-pulse stimulation was monitored for ca. 24 h prior to, and 1 week after MK801-treatment, were performed. PS amplitude and fEPSP were analyzed in both conditions. No differences in PS amplitude or in fEPSP (*n* = 6, **Figures 1A–C**) were found in animals that were injected with MK801 1 week prior to the experiment, as compared to control baseline. Thus, MK801 does not elicit long-term effects on basal synaptic transmission.

### A LONG-TERM DISRUPTION OF LTP, WHICH LASTS FOR AT LEAST 4 WEEKS OCCURS FOLLOWING ACUTE TREATMENT WITH AN IRREVERSIBLE NMDAR ANTAGONIST

**FIGURE 2:**
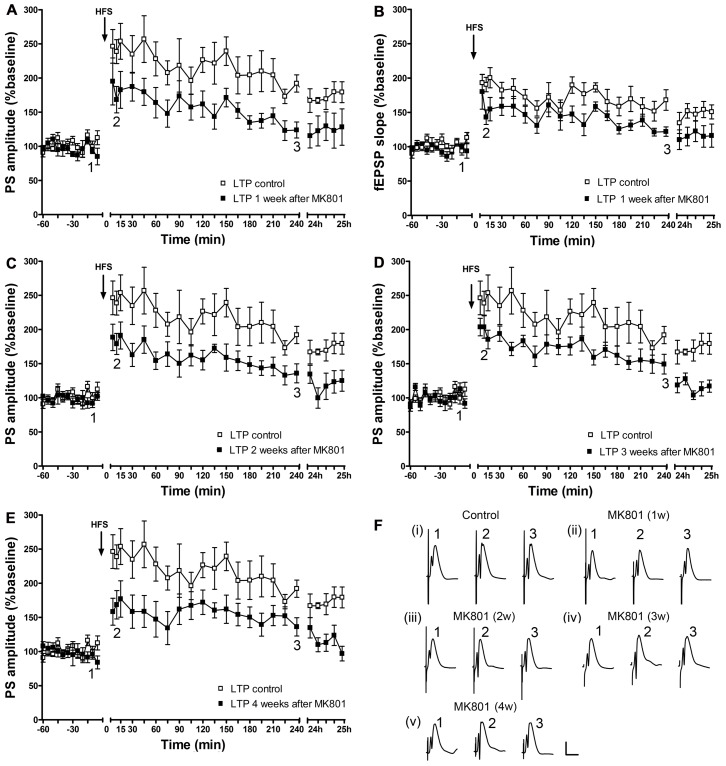
**Acute treatment with an irreversible NMDAR antagonist leads to a persistent long-term impairment of LTP *in vivo*, which lasts for at least 4 weeks.** One week after MK801-treatment, LTP of population spike amplitude **(A)** and of fEPSP **(B)** are significantly impaired compared to control LTP (*n* = 7). This impairment persists in the second (*n* = 7) **(C)**, third (*n* = 7) **(D)**, and fourth (*n* = 7) **(E)** weeks after MK801-treatment (relative changes in fEPSP responses were equivalent to changes in PS amplitude. Representative example is shown in **Figure 2B**). Line breaks on the *x*-axis indicate change in time-scale. **(F)** Original analog traces show field potentials evoked from the dentate gyrus during (i) control LTP and (ii) LTP elicited 1 week after a single systemic MK801-treatment, (iii) LTP induced 2 weeks after MK801-treatment, (iv) LTP elicited 3 weeks after MK801-treatment and (v) LTP induced 4 weeks after MK801-treatment. Vertical scale-bar corresponds to 5 mV, horizontal scale-bar to 10 ms.

One, two, three, or four weeks after an acute injection with MK801, the ability to express LTP was significantly impaired 1 week after MK801-treatment [PS: *F*(1,6) = 7.960, *p* < 0.05, *n* = 7, **Figures 2A,F**; fEPSP: *F*(1,6) = 8.940, *p* < 0.05, *n* = 7, **Figures 2B,F**]; 2 weeks [PS: *F*(1, 6) = 10.272, *p* < 0.05, *n* = 7, **Figures 2C,F**; fEPSP: *F*(1,6) = 3.047, n.s., *n* = 7, not shown], 3 weeks [PS: *F*(1,6) = 7.591, *p* < 0.05; *n* = 7, **Figures 2D,F**; fEPSP: *F*(1,6) = 11.306, *p* < 0.05, *n* = 7, not shown], and 4 weeks [PS: *F*(1,6) = 11.420, *p* < 0.05, *n* = 7, **Figures 2E,F**; fEPSP: *F*(1,6) = 7.417, *p* < 0.05, *n* = 7, not shown] after treatment with MK801, LTP was still significantly disrupted as compared to control LTP.

Thus, the MK801-induced impairment in the ability to express hippocampal LTP lasts for at least 4 weeks.

**FIGURE 3:**
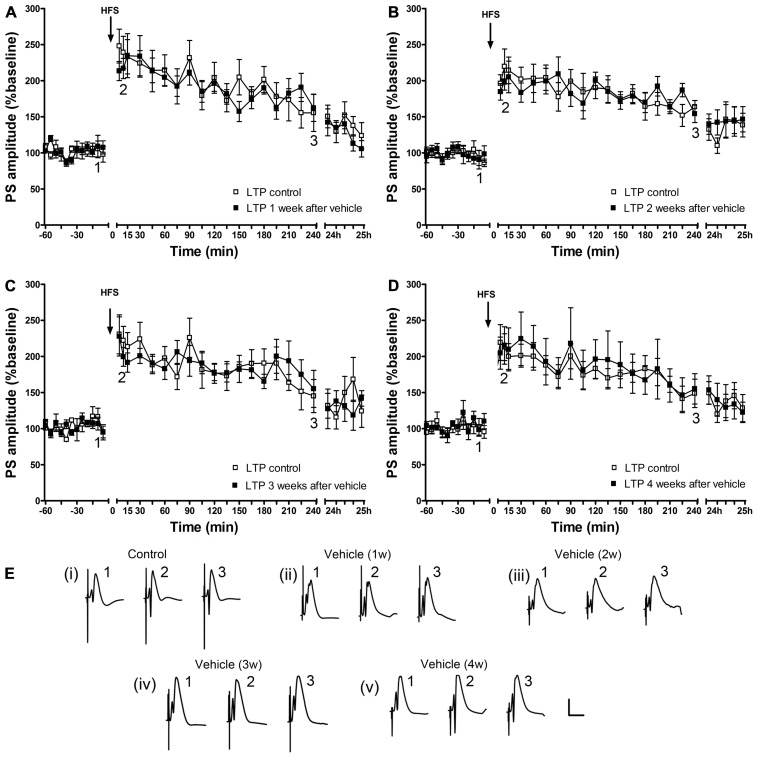
**A single vehicle injection does not change the ability to express LTP over time.** One (*n* = 6) **(A)**, 2 (*n* = 6) **(B)**, 3 (*n* = 5) **(C)** and 4 (*n* = 6) **(D)** weeks after a single vehicle injection, LTP of PS amplitude (black squares) and of fEPSP (data not shown) is not different from control LTP (white squares). Line breaks on the x-axis indicate change in time-scale. **(E)** Original analog traces show field potentials evoked from the dentate gyrus during (i) control LTP and (ii) LTP elicited 1 week after a single systemic vehicle treatment, (iii) LTP induced 1 weeks after vehicle treatment, (iv) LTP elicited 3 weeks after vehicle treatment and (v) LTP induced 4 weeks after vehicle treatment. Vertical scale-bar corresponds to 5 mV, horizontal scale-bar to 10 ms.

By contrast, in the control cohort, that was systemically treated with vehicle, the ability to express LTP was not altered (**Figure 3**).

### ONE WEEK AFTER ACUTE TREATMENT WITH AN IRREVERSIBLE NMDAR ANTAGONIST, ANIMALS DO NOT DISPLAY ALTERATIONS IN LOCOMOTOR ACTIVITY OR ANXIETY-RELATED BEHAVIOR IN THE OPEN FIELD TEST

**FIGURE 4:**
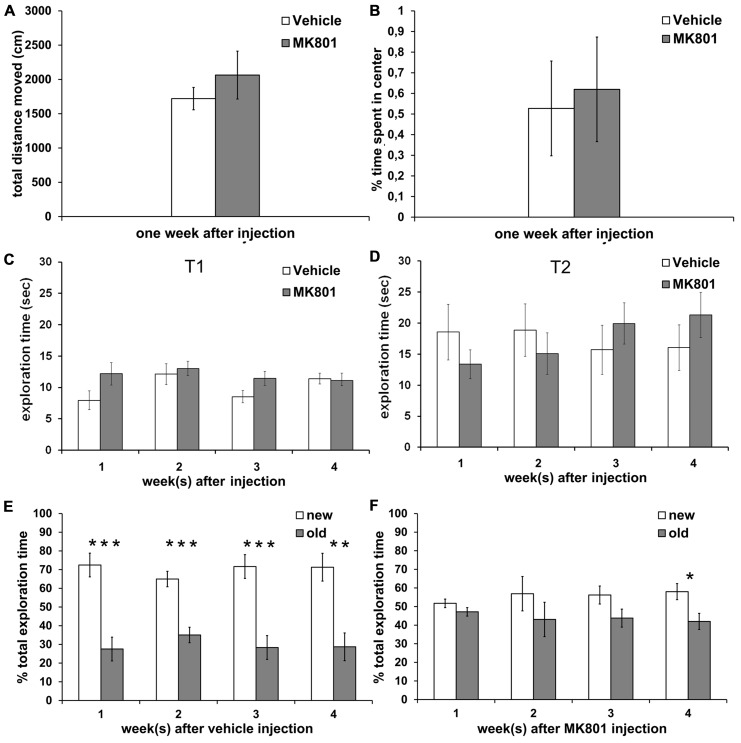
**Acute treatment with an irreversible NMDA antagonist disrupts object recognition memory for several weeks after treatment.** Locomotor activity and anxiety-related behavior are not affected. **(A)** Locomotor activity is not different between rats treated with vehicle (white column, *n* = 7) or MK801 (gray column, *n* = 7) 1 week prior to the experiment. **(B)** Vehicle (white column, *n* = 7) and MK801-injected rats (gray column, *n* = 7) spend an equal % of time in the center of the open field arena one week after treatment. **(C,D)** Total exploration times do not differ significantly between the vehicle- (white column) and MK801-treated (gray column) rats during T1 (first exposure to two new objects) and during T2 (exposure 4 h later to one familiar object and one novel object). **(E)** Control rats remember the familiar object after an interval of 4 h, as indicated by significantly longer exploration of the new (white column) as compared to the old (gray column) object at any time-point (*n* = 10 in first week, *n* = 10 in second week, *n* = 9 in third week, *n* = 9 in fourth week). **(F)** A single MK801-injection leads to a long-term impairment in object recognition memory performance which lasts for at least 3 weeks after treatment. 1 (*n* = 11), 2 (*n* = 13) or 3 weeks (*n* = 12) after an acute MK801-induced psychosis-related event, rats are not able to distinguish the familiar (gray column) from the new (white column) object, as indicated by approximately equal exploration times. In the 4 week (*n* = 13) after MK801-treatment memory performance is significant but still tends to be poorer than the performance seen in controls. Values represent means ± S.E.M.; *p < 0.05, **p < 0.001, ***p < 0.0001.

The total distance moved in the open field arena was not different between vehicle- (*n* = 7) and MK801-injected (*n* = 7) rats 1 week after treatment (**Figure 4A**). Thus, subsequent behavioral experiments were not biased by differences in locomotor abilities. In addition, both groups spent a comparable amount of time in the center of the arena (**Figure 4B**), indicating that vehicle- and MK801-treated animals did not differ in anxiety-related behavior 1 week after treatment.

### PERFORMANCE IN AN OBJECT RECOGNITION TASK IS PERSISTENTLY IMPAIRED FOLLOWING ACUTE TREATMENT WITH AN IRREVERSIBLE NMDAR ANTAGONIST

To exclude that locomotor differences could affect the outcome of behavior in the memory paradigm, we first assessed absolute exploration levels between MK801-treated and vehicle-treated animals (**Figures 4C,D**). We found no significant differences in absolute exploration levels between the different groups during the T1 (**Figure 4C**) where two identical objects were explored and T2 where a new object was substituted for one of the old objects (**Figure 4D**), either 1, 2, 3, or 4 weeks after vehicle or MK801-treatment.

Control animals demonstrated a significant memory performance 1, 2, 3, and 4 weeks after vehicle treatment in T2 (**Figure 4E**). Here, 4 h after T1 one of the now familiar objects was replaced with a novel object and exploration of both, the old and new objects, was compared.

Statistical analysis revealed significant effects at: 1 week [*t*(18) = 4.999, *p* < 0.0001; *n* = 10; **Figure 4E**]; 2 weeks [*t*(18) = 5.113, *p* < 0.0001; *n* = 10; **Figure 4E**]; 3 weeks [*t*(16) = 4.810, *p* < 0.0001; *n* = 9; **Figure 4E**] and 4 weeks after vehicle-treatment [*t*(16) = 4.053, *p* < 0.001; *n* = 9; **Figure 4E**].

By contrast, if rats underwent a single MK801-induced psychosis-like episode, they showed impaired object recognition memory during T2 when assessed 1, 2, and 3 weeks after treatment with MK801, as exploration levels for the new object were not different from those for the old object (**Figure 4F**). By 4 weeks after a single MK801-treatment, the animals showed a significant memory performance (*t* (24) = 2.616, *p* < 0.05; *n* = 13; **Figure 4F**), but tended to be less able to distinguish the new object from the old compared to the performance of the control animals. For example, control animals explored the new object ca. 70% of the time 4 weeks after vehicle-treatment, whereas MK801-treated animals explored the new object less than 60% of the time.

### CELL NUMBERS WITHIN THE RSC, DG, AND EC DID NOT DIFFER BETWEEN CONTROLS AND MK801-INJECTED ANIMALS 4 WEEKS AFTER TREATMENT

**FIGURE 5:**
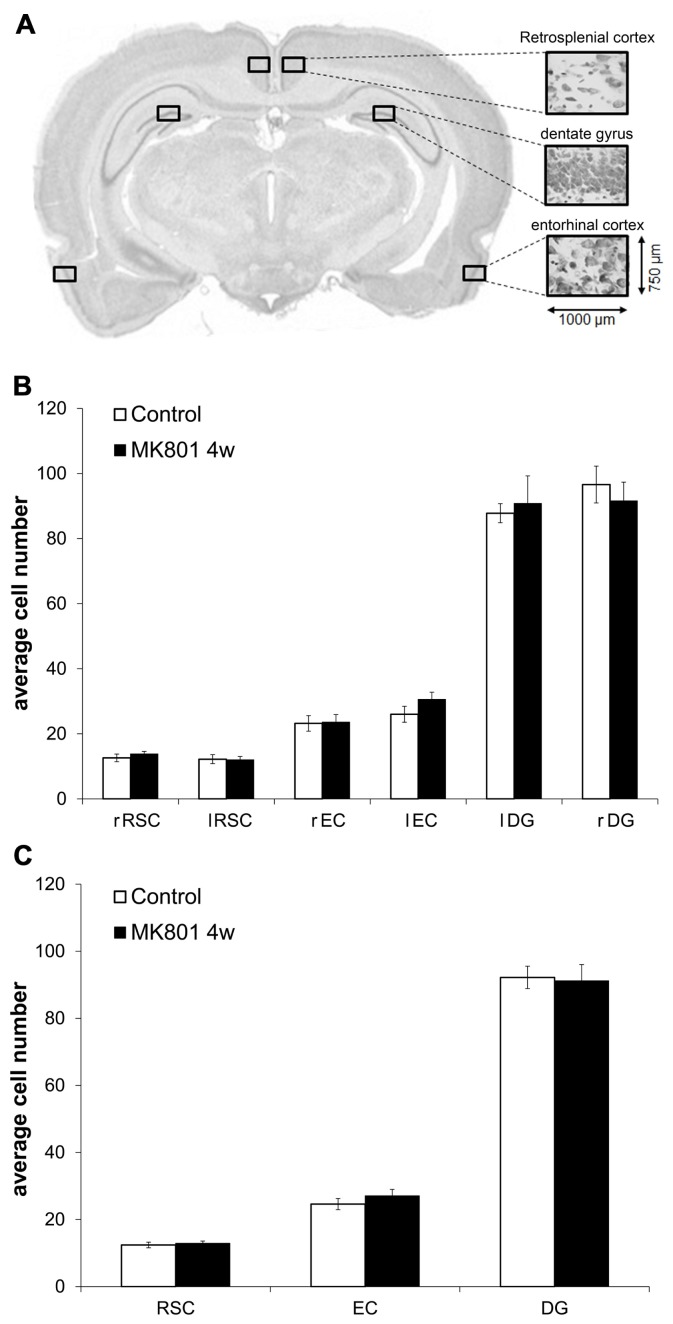
**Neuron numbers do not differ in the retrosplenial cortex (RSC), dentate gyrus (DG), and entorhinal cortex (EC) when tissue from vehicle- and MK801-injected animals is compared 4 weeks after treatment. (A)** Representative example of the section plane analysed (-5.3 respect to Bregma) with the predefined regions of interest (ROIs) of the same size (750 μm × 1000 μm) in the left and right hemispheres. **(B,C)** Average cell numbers do not differ between controls and MK801-treated animals 4 weeks after injection. RSC, retrosplenial cortex; DG, dentate gyrus; EC, entorhinal cortex; r, right; l, left.

MK801 has been reported to induce neurodegeneration at high concentrations (typically 10 mg/kg) in certain brain areas, primarily in the RSC (Olney et al., 1989). In order to investigate if our concentration may have caused degenerative effects, we performed cell counts in the RSC, the DG, and the EC. The number of viable neurons did not differ between MK801- and vehicle-treated animals in any of the analyzed regions 4 weeks after treatment (**Figures 5B,C**).

## DISCUSSION

This study shows that acute, single treatment with an irreversible NMDA receptor antagonist elicits lasting effects on hippocampal function: both LTP and object recognition memory are profoundly impaired, with effects lasting for weeks. This finding is striking, as it suggests that NMDA receptor hypofunction occurs as a consequence of treatment. This may take the form of a lack of recovery of receptor numbers, but could also derive from changes in NMDA receptor subunit constellations and /or distributions as a result of MK801 application. Evidence of the latter, following subchronic MK801-treatment has been reported (Wang and Gao, 2012). NMDA receptor hypofunction is believed to contribute to schizophrenia-spectrum disorders including psychosis (Coyle et al., 2003; Vrajová et al., 2010). Our current observations therefore open up the interesting possibility that the cognitive deficits that accompany schizophrenia in psychotic patients may derive from NMDA receptor dysfunction and an associated a loss of hippocampus-dependent synaptic plasticity and learning.

Irreversible uncompetitive NMDAR-antagonists induce psychotic symptoms in healthy humans and exacerbate symptoms of schizophrenic patients (Krystal et al., 1994; Lahti et al., 2001). Out of all known NMDAR-antagonists, MK801 (dizocilpine) has the highest potency for the phencyclidine (PCP) binding site and strongest psychotomimetic properties (Kornhuber and Weller, 1997). Immediately following treatment with the irreversible NMDA receptor antagonist, MK801 rodents exhibit symptoms of acute psychosis (Wöhrl et al., 2007; Manahan-Vaughan et al., 2008a,b). Recovery is rapid but is accompanied by plasticity deficits, and it is tempting to speculate that this state emulates first-episode psychosis that typically heralds the onset of chronic schizophrenia in humans (McGlashan, 2006; Alvarez-Jimenez et al., 2011). An unanswered question is what mechanisms underlie the transition from first-episode psychosis to chronic schizophrenia? Our results reveal that a single psychosis-like episode in rodents produces persistent long-term consequences, in the areas of cognition and synaptic plasticity. We hypothesize that these enduring cellular consequences might mirror an increased overall vulnerability of the brain after acute psychosis, that in turn reflects physiological adaptations that enable the establishment of the disease.

In our behavioral experiments, long-term deficits in object recognition memory were detected for at least 3 weeks after acute treatment with MK801. The ORT is widely used for assessment of memory deficits in animal models of schizophrenia (de Lima et al., 2005; Goulart et al., 2010; Snigdha et al., 2010). The rationale behind the use of this behavioral procedure is the fact that schizophrenic patients show profound deficits in the recognition and recall of non-verbal stimuli (Schretlen et al., 2007). Moreover, unlike other animal memory tests (e.g., radial maze, Morris water maze, or T-maze), the ORT does not require motivational factors such as e.g., food reward or escape from water. It is purely based on the intrinsic motivation of the rodent, which makes it more valid in terms of its comparability to human memory tests. It has been already shown that rats treated with an irreversible NMDAR-antagonist display impaired performance in the ORT immediately after treatment (Nilsson et al., 2007; van der Staay et al., 2011). However, the duration of this effect has not yet been investigated systematically over such a long period. The fact that the cognitive symptoms were specifically and persistently affected after MK801-treatment is in line with the widespread belief that cognitive deficits, such as impairments in declarative memory, dominate as factors leading to poor functional outcomes in schizophrenia (Stone and Hsi, 2011).

Another major finding of our study is the fact that hippocampal synaptic plasticity is the most prominently and potently affected parameter after acute MK801-treatment and is therefore putatively the central long-term consequence of NMDA receptor hypofunction. Our results show that the inability to express normal LTP in the hippocampus persists for at least 4 weeks after a single MK801-treatment. Hippocampal LTP is a widely studied cellular phenomenon, comprising strengthening of synaptic efficacy after repetitive stimulation, which is believed to underlie learning and memory (Bliss and Collingridge, 1993). The concept of synaptic plasticity has been linked to schizophrenia pathology in many contemporary theoretical considerations (Peled, 2005; McGlashan, 2006). For instance, the question, as to whether the process mediating consequences of active psychosis might comprise neurotoxicity or rather impaired synaptic plasticity, was introduced just a few years ago (McGlashan, 2006). However, as neuronal death and reduction in neuron numbers has not been convincingly demonstrated in schizophrenic post-mortem tissue (Harrison, 1999), whereas reduced spine densities (Garey et al., 1998; Glantz and Lewis, 2000) were reported, the synaptic plasticity hypothesis has become regarded as the more plausible one. In addition, many of the identified schizophrenia susceptibility genes have been linked to synaptic plasticity (Harrison and Weinberger, 2005). In line with these findings, our study experimentally supports the assumption that impaired ability to express synaptic plasticity is the main pathological factor underlying acute psychosis. Furthermore, we hypothesize that impaired synaptic plasticity is the cellular substrate of increased vulnerability for further psychotic episodes caused by future stressful events.

Although the impairment in the ability to express LTP correlates strongly with deficits in memory, it outlasts the effects on memory observed in this study, which is probably due to the relative insensitivity of the behavioral test procedure. In any case, synaptic plasticity seems to be the most representative measurement of persistent long-term effects after a single psychotic episode. Therefore it might be the most valuable and valid parameter for testing potential new therapeutics in preclinical settings. However, the extrapolation from animal to human should always be considered with reservation, especially, in psychiatric research. Of course, the question as to whether synaptic plasticity is also affected in humans in a long-term manner following first-episode psychosis remains speculative. Nevertheless, recent evidence indicates that LTP-like facilitation is indeed inducible in humans via e.g., TMS (Korchounov and Ziemann, 2011) and that LTP may be disrupted in schizophrenic patients, albeit in another cortical area (Frantseva et al., 2008).

In any case, the mechanism underlying this persistent effect remains to be discovered. We hypothesize that a single treatment with MK801 induces a long-lasting physiological change of the whole system which will probably be reflected by an enduring NMDAR-hypofunction. This hypofunction does not necessarily relate to the NMDAR numbers, as MK801 (bound to NMDAR) will be metabolized from the brain (Wegener et al., 2011) and new NMDARs will be inserted into the membrane to replace those lost. It may also result from altered NMDAR subunit expression, or from a dysfunctional molecule up- or downstream to the NMDAR itself. These possibilities should be the subject of future studies.

MK801 at high concentrations can induce irreversible damage in a brain area and gender-specific manner with the RSC in females being the most vulnerable (Olney et al., 1989; Bender et al., 2010). Specifically, at the concentration used in this study (5mg/kg), several studies found neurotoxic effects in certain brain areas (Fix et al., 1993; Farber et al., 1995), others found partly transient damage (Horváth et al., 1997) or even failed to detect any morphological changes at all after MK801-treatment (Wöhrl et al., 2007). Our quantitative analysis of viable cell numbers within the DG, EC, and RSC revealed no neurotoxic effects 4 weeks after MK801-treatment as compared to controls. Thus, either MK801 did not induce degeneration at the concentration used, or possible MK801-induced effects on cell numbers were transient and therefore undetectable 4 weeks after treatment. Hence, we cannot completely rule out the possibility that potential neurotoxic effects elsewhere in the brain may contribute to the effects observed in this study. However under the conditions used, we did not find evidence of brain damage and therefore it is unlikely that neurotoxicity played a role in our results.

Pharmacological animal models of psychosis are usually utilized for assessment of potential therapeutic effects of new compounds. The model of acute psychosis used in this work has already been implemented in such preclinical studies. For example, 1 week after MK801-treatment the deficit in LTP can be prevented by different substances such as an inhibitor of the glycine transporter-1 (GlyT1) or inhibition of the phosphodiesterase-4 (PDE4) (Manahan-Vaughan et al., 2008b; Wiescholleck and Manahan-Vaughan, 2012). The current finding, that in this model the deficits in LTP and in object recognition memory persist for weeks, opens up the possibility of exploration of the duration of the therapeutic effects of test compounds.

Finally, in line with numerous previous studies (Harrison, 2004; Heckers and Konradi, 2010; Tamminga et al., 2010) this finding supports a pivotal role of the hippocampus in the pathology of schizophrenia-spectrum disorders. Of note, recently, hippocampal LTP has been shown to profoundly influence prefrontal cortex activation (Canals et al., 2009) – hence, it is thinkable that MK801-induced long-term impairment in synaptic plasticity in the hippocampus will affect and spread to other schizophrenia-relevant brain structures. A graph theory model of NMDA receptor hypofunction in schizophrenia predicts that this may indeed be the case (Dawson et al., 2012).

## CONCLUSION

Taken together, our data suggest that an acute psychosis-like episode, induced by a single treatment with MK801, produces long-term effects on the synaptic and cognitive behavioral levels. Thus, this might reflect a mechanism by which an acute psychotic event supports the development of chronic illness. As the inability to express normal synaptic plasticity persists for the longest period, we propose that deficits in hippocampal LTP may comprise one of the cellular substrates leading to increased vulnerability of the brain towards developing further psychotic episodes.

## Conflict of Interest Statement

The authors declare that the research was conducted in the absence of any commercial or financial relationships that could be construed as a potential conflict of interest.

## Abbreviations

ANOVA: analysis of variance
DG: dentate gyrus
EC: entorhinal cortex
fEPSP: field excitatory postsynaptic potential
GlyT1: glycine transporter-1
HFS: high frequency stimulation
i.p.: intraperitoneal
LTP: long-term potentiation
NMDA: *N*-methyl-D-aspartic acid or *N*-methyl-D-aspartate
NMDAR: *N*-methyl-D-aspartic acid or *N*-methyl-D-aspartate receptor
ORT: object recognition task
PCP: phencyclidine
PDE4: phosphodiesterase 4
PS: population spike
RSC: retrosplenial cortex
ROI: region of interest
T1: trial 1
T2: trial 2
TMS: transcranial magnetic stimulation.
